# Interaction Effect of Food Insecurity and Stroke on the Risk of All-Cause Mortality: NHANES 2015–2018

**DOI:** 10.3390/foods14132281

**Published:** 2025-06-27

**Authors:** Sri Banerjee, W. Sumner Davis, Jagdish Khubchandani, Patrick Dunn

**Affiliations:** 1 College of Health and Public Health, Walden University, Minneapolis, MN 55401, USA; sumner.davis@mail.waldenu.edu (W.S.D.);; 2 College of Health, Education and Social Transformation, New Mexico State University, Las Cruces, NM 88003, USA; jagdish@nmsu.edu; 3 American Heart Association, Dallas, TX 75231, USA

**Keywords:** stroke, NHANES, food insecurity

## Abstract

**Background**: Stroke continues to be a major cause of morbidity mortality in the United States. In this study, we determined if the food insecurity status interacted with a history of stroke to influence the overall mortality risk. **Methods**: Data from the 2015–2018 National Health and Nutrition Examination Survey, a nationally representative survey among the non-institutionalized population that is published in two-year cycles, with a mortality follow-up through 31 December 2019 was analyzed in this investigation. **Results**: In stroke survivors, upon follow-up, a higher proportion of those with food insecurity died (38.1% vs. 31.6%, *p* < 0.05) than food-secure individuals. For overall mortality, the crude hazard ratio (HR) for stroke survivors was 5.87 (95% confidence interval [CI], 3.18–10.86, *p* < 0.01). After adjustment for multiple variables, the HR was significantly elevated, 3.66 (CI 1.64–8.14, *p* < 0.01), among stroke survivors with food insecurity, but among those with stroke only or just food insecurity, the HR was not significantly elevated. Similar interactions were seen among females but not among males. **Conclusions**: Food insecurity substantially increases mortality from all causes among stroke survivors. When considering various chronic diseases, such as stroke, the role of social problems must be taken into consideration.

## 1. Introduction

The Food Agriculture Organization (FAO) defines food security as “when all people, at all times, have physical, social, and economic access to sufficient, safe, and nutritious food that meets their dietary needs and food preferences for an active and healthy life [1]”. The FAO definition provides more specificity to the access component of food insecurity by describing quality food and endorsing specific needs and preferences. Also, the FAO mentions that food security is responsible for normal growth and development and an active and healthy life [1,2]. A lack of food security can lead to stress, increased allostatic load, the risk of acute and chronic health problems, and poor disease outcomes [3,4,5]. In the U.S., the rates of food insecurity continue to remain higher than many peer nations and also vary according to sociodemographic characteristics, with the highest proportion of food insecurity seen among those who live in poverty, have lower education, or live in rural or southern regions [5,6,7]. Also, food insecurity can be temporary or chronic and affects individuals, families, and communities, often driven by food deserts, rising food prices, or natural and economic disruptions. In the last ten years, food insecurity rates in some geographic areas across the U.S. have affected more than a fifth of the population in these areas that are dominated by vulnerable and marginalized groups, such as racial and ethnic minorities [5,6,7,8].

The presence of social maladies makes the individual more vulnerable to the effects of chronic disease. Specifically, food insecurity is associated with chronic diseases, such as heart disease, diabetes, kidney diseases, and hypertension [6,7,8,9,10]. Stroke, a leading cause of mortality, is closely intertwined with social factors such as food insecurity. Stroke survivors who experience food insecurity often belong to marginalized communities that already face systemic healthcare disparities. The National Center for Health Statistics reported that non-Hispanic Black and Hispanic populations have higher rates of food insecurity and disproportionately suffer from poor stroke outcomes. These disparities are exacerbated by limited healthcare access, financial constraints, racism, and educational barriers that reduce awareness of proper nutrition and disease management [7,8,9,10].

Prior research has shown that food insecurity is associated with higher mortality rates among those with chronic and cardiovascular diseases, suggesting that limited access to nutritious food exacerbates health complications among those with cardiovascular or cerebrovascular diseases [11,12,13]. Food insecurity affects multiple pathways contributing to poor stroke outcomes, including inadequate nutrition, increased psychological stress, and reduced access to healthcare services [13,14,15]. Recently, there has been a lot of interest in the association between food insecurity and acute cerebrovascular and cardiovascular events, with some scholars suggesting that food insecurity could be among a common and frequently overlooked social factor [14,15,16]. Specifically, for brain health, a large systematic review included 30 studies reporting that food insecurity impacts brain health in various ways such as decreased cognition and poor mental health [17]. Another recent systematic review of 30 studies found that food insecurity is associated with poor cognition and worsened psychiatric outcomes [18]. A major meta-analysis of 123 studies found that various types of diets such as whole grains, vegetables, fruits, nuts, legumes, dairy, fish, red and processed meat, eggs, and sugar-sweetened beverages are linked with stroke risk [12]. Despite, these studies, there is a paucity of evidence on how food insecurity affects survival among stroke survivors after accounting for various health and sociodemographic factors. Thus, the purpose of this study was to assess the influence of food insecurity on survival rates among stroke survivors from a large national random sample of adult Americans.

## 2. Methods

### 2.1. Study Participants and Procedures

For this study, we employed data from the 2015–2018 cycles of the National Health and Nutrition Examination Survey (NHANES), which is administered by the National Center for Health Statistics (NCHS), USA [3,5,14]. The data used in this study can be accessed at https://pubmed.ncbi.nlm.nih.gov/33663649/ (accessed on 13 November 2020) and https://wwwn.cdc.gov/nchs/nhanes/continuousnhanes/default.aspx?BeginYear=2015 (accessed on 13 November 2020). This survey, which is representative of the national population, is conducted annually to evaluate the health and nutritional status of adults in the U.S., utilizing integrated data obtained from standardized interviews and physical examinations. Our analysis is limited to a sample of adults aged 20 years and higher of non-institutionalized U.S. adults. The mortality status was examined by linking NHANES participant survey records with publicly accessible mortality data from the National Death Index (NDI) [3,5,14,19,20]. The NCHS identified all-cause mortality through probabilistic matching techniques between NHANES respondents and NDI death certificate records, and all procedures and protocols for these publicly available datasets were approved by NCHS ethics boards. We obtained additional ethical approval from the Walden University IRB for the analysis of data using publicly available files.

### 2.2. Measures

#### 2.2.1. Outcome Variable

All-Cause Mortality. The vital status was assessed utilizing the Continuous NHANES Public-Use Linked Mortality File from the National Death Index. This dataset offers follow-up information on participant vital status, measured in person-months, beginning from the date of survey participation and extending through either the recorded date of death or 31 December 2019, whichever occurred first. Additional details about the analysis and study design for these variables and NHANES are published elsewhere [14,19,20].

#### 2.2.2. Predictor Variables

To assess food insecurity, we utilized the USDA Household Food Security Survey Module (HFSSM), a rigorously validated instrument designed to evaluate household food security status over the preceding 12 months [3,4,5,6]. For this study, 10 adult-relevant HFSSM items were utilized in the analysis (i.e., items pertaining to children were excluded). Initially, food insecurity was categorized into four levels: food secure, marginally food secure, low food secure, and very low food secure. Subsequently, HFSSM responses were dichotomized using established cutoff points: individuals were classified as food secure if they responded negatively to all scale items [3,4,5]. Participants who responded affirmatively to one or more items were classified as food insecure, encompassing marginal, low, and very low food security levels. Sensitivity analyses confirmed the robustness of the results across both dichotomous and 4 level classification models of food insecurity. Those who answered “yes” to the following question: “Have you ever been told by a physician or a health professional that you had a stroke?” were considered to have a stroke history [13,14,15,16].

#### 2.2.3. Key Health-Related and Demographic Variables

We used the standard variables as confounders similar to previous studies from the NHANES database. The chronic kidney disease (CKD) status, derived from clinical laboratory measures, was determined using serum creatinine levels in accordance with the Chronic Kidney Disease Epidemiology Collaboration (CKD-EPI) equation, developed by Levey and colleagues. The CKD-EPI equation is expressed as

eGFR cr = 142 × min(Scr/κ, 1)^α × max(Scr/κ, 1) − 1.209 × 0.993^Age × 1.012 [if female],

where Scr represents serum creatinine, κ is 0.7 for females and 0.9 for males, and α is −0.329 for females and −0.411 for males. The min and max functions represent the minimum and maximum of Scr/κ or 1, respectively. Participants with an albumin-to-creatinine ratio (ACR) greater than 30 mg/g or an estimated glomerular filtration rate (eGFR) below 60 mL/min/1.73 m^2^ were categorized as having CKD, based on an expanded clinical definition. Hypertension was determined based on a survey question where yes was considered confirmation for “Have you ever been told by a physician or a health professional that you have hypertension?”

Body mass index (BMI), calculated from the measured height and weight, was classified into four categories: normal weight (BMI < 25 kg/m^2^), overweight (BMI 25–29.9 kg/m^2^), obese (BMI 30–39.9 kg/m^2^), and severely obese (BMI ≥ 40 kg/m^2^). For multivariate analyses, BMI was used as the continuous variable. Smoking status was determined using a two-variable indicator: participants were categorized as “smokers” if they reported having smoked at least 100 cigarettes in their lifetime and answered “every day” or “some days” to the question, “Do you now smoke cigarettes?” Otherwise, individuals were categorized as “nonsmokers.” Participant characteristics such as age, sex, education, marital status, and race/ethnicity were assessed for this analysis. The FIPR (family income to poverty ratio) was considered as an indicator of poverty/income status.

### 2.3. Statistical Analysis

We weighted demographic variables to approximate distributions in the U.S. by using the provided sample weights (to account for oversampling of certain groups, unequal probabilities of selection, and non-responses). Categorical variables were expressed as percentage values and analyzed using chi-square tests. Complex samples multiple Cox regression models were used to examine the risk of all-cause mortality based on food insecurity or stroke after adjusting for covariates. Statistical analyses were conducted using the SAS v9.5 and SUDAAN 11.0.4 software. Additionally, we generated survival curves showing the combined effect of stroke and food insecurity using the Kaplan–Meier product-limit method to estimate the percent survival of the subject at each point in time.

## 3. Results

A total of 16,308 adults were included in the final sample where more than a tenth had had food insecurity (16.3%) and less than one in twenty (4.0%) reported a stroke history. The average duration of follow-up for all study participants was 10.7 years. Table 1 provides data on the distribution of demographic characteristics of the study participants stratified by stroke using bivariate analysis. Individuals with stroke were statistically significantly more likely to be older, with lesser education and income, racial/ethnic minorities, and widowed/divorced. Also, individuals with stroke were significantly more likely to report food insecurity or hypertension or have obesity or CKD.

**Figure 1:**
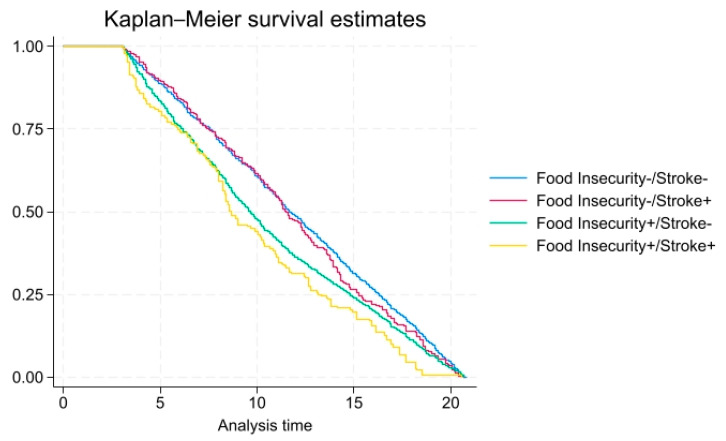
All-cause mortality among adults with and without food insecurity/stroke (x-axis: time in years, y-axis: overall survival probability).

In Table 2, for overall mortality, compared to those without stroke, the crude hazard ratio (HR) among those with a stroke history was 5.87 (95% confidence interval [CI], 3.18–10.86, *p* < 0.01) in the tables). The adjusted HR was elevated, 3.66 (CI 1.64–8.14, *p* < 0.01), among stroke patients with food insecurity but close to 1.0 (0.87 CI 0.39–1.94, *p* = 0.60) in stroke patients with food security, after adjusting for health and demographic variables. As shown in Figure 1, there was a higher probability of all-cause mortality over time (mean = 10.7 years) among individuals with both stroke and food insecurity combined.

**Figure 2:**
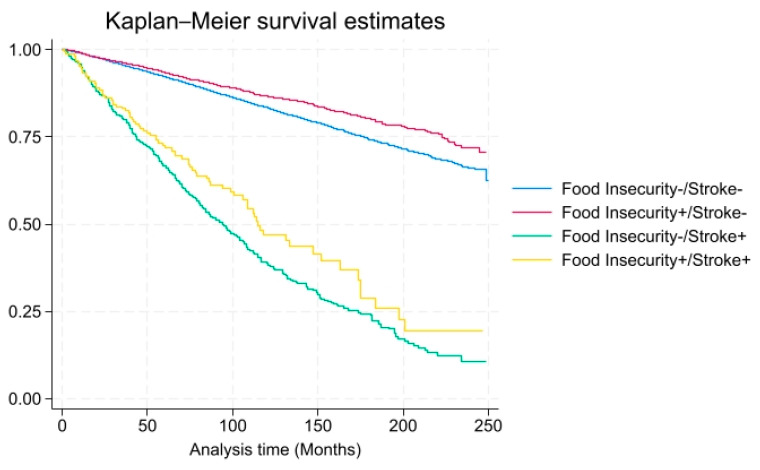
All-cause mortality among males with and without food insecurity/stroke (x-axis: time in months, y-axis: overall survival probability).

**Figure 3:**
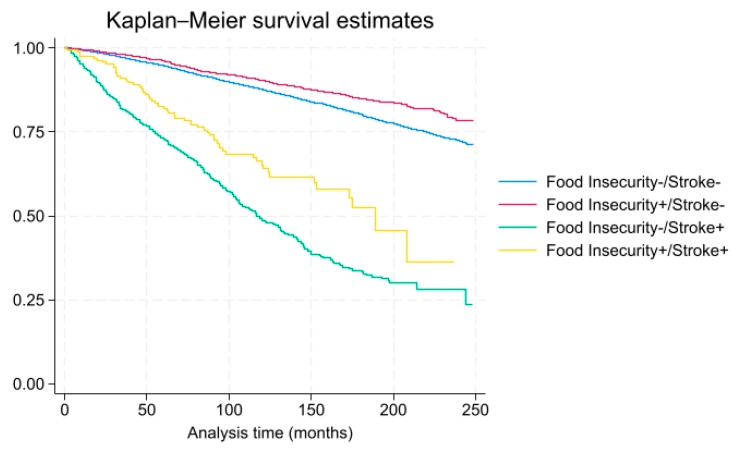
All-cause mortality among females with and without food insecurity/stroke (x-axis: time in months, y-axis: overall survival probability).

As shown in Table 3 and Table 4, when stratified by gender, among males, the adjusted HR was not significantly elevated, 7.20 (CI 0.73–70.78, *p* = 0.50), among stroke patients with food insecurity and close to 1.0 (0.74 CI 0.30–1.85, *p* = 0.40) in stroke patients with food security, after adjusting for health and demographic variables. However, among females, the adjusted HR was significantly elevated, 8.76 (CI 1.36–56.49, *p* < 0.01), among stroke patients with food insecurity but close to 1.0 (1.36 CI 0.45–4.13, *p* = 0.60) in stroke patients with food security, after adjusting for health and demographic variables. As shown in Figure 2 and Figure 3, males and females with stroke had increased mortality and food insecurity influenced this risk. As shown in there was a higher probability of all-cause mortality over time among individuals with food insecurity and stroke.

## 4. Discussion

To our knowledge, this was the first large nationally representative study to show that there is an association between stroke and food insecurity as it relates to mortality risk. Several studies have established a correlation between food insecurity and increased risk for cardiovascular diseases [20,21,22,23]. A recent NHANES data study highlighted that individuals experiencing food insecurity are more likely to have risk factors such as hypertension, diabetes, and obesity, all of which contribute to stroke incidence [5]. Another NHANES study demonstrated that food insecurity leads to an increased risk of all-cause mortality and cardiovascular mortality [14]. The physiological impact of food insecurity can lead to increased stress, dietary deficiencies, and heightened inflammatory responses, further exacerbating stroke risk [24,25]. When resources are limited, people often turn to affordable, calorie-rich foods that are high in saturated fats, refined sugars, sodium, fiber, vitamins, and minerals. These foods include instant meals, processed meats, sugary and salty snacks, or beverages. These dietary patterns have also been linked with a higher risk of stroke and poor cardiovascular health [23,24,25,26].

Certain diets and eating patterns have been shown to result in the vicious cycle of poor cardiovascular health and food insecurity. Due to cost, there is a reduction in the intake of heart-healthy foods. Food-insecure households consume less fresh produce, whole grains, lean proteins, and healthy fats (like those from nuts, seeds, or olive oil)—all critical for cardiovascular health [25,26,27,28]. Cyclical eating patterns (feast or famine) can generate disrupted eating schedules (e.g., overeating when food is available and undereating when food is unavailable), which contribute to metabolic stress, insulin resistance, blood pressure fluctuation, chronic stress, and coping mechanisms (HPA Axis Stimulation) and can create psychosocial stress, which is a risk factor in itself for cardiovascular disease. Stress may also drive emotional eating, often of unhealthy comfort foods [24,25,26,27,28,29,30,31,32].

From this large multi-ethnic population, we found that stroke survivors with food insecurity had significantly higher all-cause mortality rates than their food-secure counterparts even after controlling for social factors such as poverty, education, and ethnic minority status. When stratified by gender we found that female stroke survivors with food insecurity had significantly higher all-cause mortality rates than their food-secure counterparts. However, the same interaction relationship was not true for males. These findings demonstrate that female stroke survivors are more prone to the impact of food insecurity than male stroke survivors. The precise mechanisms of mortality among female stroke survivors in the presence of food insecurity may need further exploration from a physiological standpoint.

We also found that several social determinants, such as poverty, education, and ethnicity are associated with higher rates of stroke. Also, food insecurity is disproportionately high among low-income populations, racial and ethnic minorities, and individuals with lower educational attainment [2,3,4,5,30,31,32,33]. The common social disparities lead to higher rates of stroke as well as food insecurity. While it is acknowledged that individuals living in deprived and lower socioeconomic status environments experience a wide array of intersecting challenges—such as housing instability, unemployment, exposure to violence, and systemic marginalization—food insecurity should not be underestimated as a contributing factor to adverse health outcomes [3,4,5,6,32,33,34,35]. Food insecurity often acts as a compounding and amplifying stressor within a broader ecosystem of disadvantage and is both a symptom and a driver of structural inequity, with direct physiological, behavioral, and psychosocial consequences [2,3,4,5,6,7,8,30,31,32,33,34].

There are indirect and direct links between mental health and social factors (e.g., food insecurity). More specifically, the psychological burden of food insecurity—characterized by chronic stress, anxiety, and loss of agency—can exacerbate other social stressors through mechanisms such as elevated cortisol levels, impaired decision-making, and reduced adherence to medical or lifestyle recommendations [5,11,17,18,32,33,34]. Therefore, while food insecurity may not singularly explain all adverse outcomes in complex contexts, it remains a significant, modifiable, and policy-relevant determinant of health that warrants careful attention in both research and intervention strategies [30,31,32,33,34,35]. Furthermore, from a biological perspective stress associated with food insecurity leads to increased allostatic load, which accelerates disease progression and may worsen post-stroke recovery outcomes. More specifically, non-Hispanic Black individuals had higher rates of stroke than those without stroke [3,4,5,6,7,8,9]. Previous researchers have shown that ethnic minority individuals have been reported to experience increased rates of chronic disease and stroke., suggesting that systemic disparities play a role in health outcomes. Moreover, economic factors, such as the poverty–income ratio (PIR), contribute to limited and nutritious food, worsening health disparities [2,3,13,15,16]

While previous studies have shown associations between food insecurity and poor outcomes, our work is novel in demonstrating a strong interaction effect—the tripling of mortality risk in patients with both stroke and food insecurity, after adjusting for key social and medical risk factors. Some researchers have attributed the phenomenon of food insecurity solely due to poverty and racial minority status. In this study, we found that food insecurity independently leads to mortality even when social disparities are taken into consideration. Stroke survivors who experience food insecurity often belong to marginalized communities that already face systemic healthcare disparities [4,6,16,21,22]. These disparities are exacerbated by limited healthcare access, financial constraints, and educational barriers that reduce awareness of proper nutrition and disease management. Food insecurity leads to increased mortality due to the compounded effects of chronic disease burden, poor access to healthcare, and inadequate nutrition [32,33,34,35,36,37].

### 4.1. Limitations

Although nationally representative surveys such as the National Health and Nutrition Examination Survey (NHANES) offer a rigorous and standardized method for assessing population-level dietary intake and health outcomes, several limitations must be acknowledged. First, despite its nationally representative design, NHANES may underrepresent certain subpopulations, including undocumented migrants, homeless individuals, and residents of institutional settings. This may limit the generalizability of findings to the broader U.S. population or to specific vulnerable groups. Second, dietary intake data in NHANES is largely based on self-reported 24 h recall methods, which are subject to recall bias and social desirability bias, potentially distorting actual consumption patterns. Furthermore, the observational nature of the survey restricts causal inferences. Also, from this research, the directionality between food security versus stroke cannot be established. However, hypothetically, the food security questionnaire was more recent (previous 12 months) than the occurrence of prevalent stroke. NHANES also may not adequately capture seasonal dietary variation, culturally specific food items, or complex nutrient interactions found in real-world eating behaviors. Additionally, while food insecurity data are typically collected at the household level, health outcome data are collected at the individual level, introducing potential analytical inconsistencies [36]. Lastly, due to the time lag between data collection and dissemination, the findings may not reflect more recent socio-economic changes such as inflation or post-pandemic shifts in food access. Consequently, while NHANES data provides a critical foundation for population health research, its findings should be interpreted with caution and ideally complemented by longitudinal and community-based studies.

While the HFSSM does not capture food composition or contamination risks, it is a validated tool widely used in public health to assess food access and availability. Due to the self-reported data in the questionnaire, there is a potential recall bias. In addition, there is also the potential for social desirability bias. While the HFSSM has been validated across populations with varying educational backgrounds, ensuring standardized administration that mitigates differential interpretation, the potential mediating effect of health literacy and health-consciousness could not be measured.

### 4.2. Recommendations

Despite the growing body of literature linking food insecurity and stroke, several gaps remain. Few longitudinal studies examine the long-term effects of food insecurity on stroke survivors. Additionally, more research is needed to explore the effectiveness of interventions—such as food assistance programs and nutritional counseling—in improving stroke outcomes. Future studies should also investigate the intersection of food insecurity with other social determinants, such as economic instability, housing instability, and healthcare access, to develop comprehensive public health strategies [31,32,33,34,35,36].

Food insecurity is increasingly recognized as a critical social determinant of health (SDoH), particularly among individuals with chronic conditions, necessitating social scientists to rethink the SDoH model to include a separate domain for food insecurity. SDoH and psychological stressors must be screened for by healthcare practitioners [21,22,23,31,32,33]. Also, targeted interventions are needed to address food insecurity as part of post-stroke care. Interventions to reduce food insecurity may significantly improve stroke outcomes and overall mortality rates. Studies have shown that participation in federal nutrition assistance programs, such as the Supplemental Nutrition Assistance Program (SNAP), is associated with improved cardiovascular health and lower mortality rates among vulnerable populations [34,35,36]. Additionally, community-based initiatives focusing on increasing access to fresh produce, nutritional education, and chronic disease management support could mitigate the negative effects of food insecurity on stroke patients [33,34,35,36,37,38,39]. Policymakers should allocate budgetary funds to safety net programs that address food insecurity, funded through an assortment of funding mechanisms, including grants, governmental funds, and donations [36,37,38,39]. There is also a need to design and implement effective messages and education concerning the role of food for brain and heart health, both affecting stroke risk. To enhance its practical value and translation, future research should focus on identifying mechanistic pathways, population-specific moderating factors, or intervention strategies that mediate the food insecurity–stroke mortality link, thereby offering deeper explanatory or real-world actionable evidence.

## 5. Conclusions

In this large nationwide study of adult Americans, we found that food insecurity increases the risk of mortality among stroke survivors (specifically, among female stroke survivors). Several moderating factors influenced this relationship between food insecurity, stroke, and survival probability (e.g., age, sex, and history of chronic diseases). These relationships could exist due to impaired nutrition and dietary deficiencies complicating chronic disease management and increasing psychological stress, both related to worsened disease outcomes (e.g., among stroke survivors). Despite the growing body of literature linking food insecurity and stroke, several gaps remain. Longitudinal studies should examine the long-term effects of food insecurity on stroke survivors and more research is needed to explore the effectiveness of interventions—such as food assistance programs and nutritional counseling—in improving stroke outcomes. Also, future research should explore the long-term impacts of diet quality, food availability, and diet patterns on stroke mortality or mortality from other causes among stroke survivors.

## Figures and Tables

**Table 1:** Characteristics of study participants stratified by stroke.

Characteristics	Total Population (*n* = 16,308)	Stroke (+) (*n* = 647)	Stroke (−) (*n* = 15,661)
Food Insecurity *	16.3 (14.5–18.0)	23.9 (16.2–31.6)	16.0 (14.3–17.7)
Smoking Status **			
Never Smoked	56.9 (55.3–58.4)	40.6 (35.6–45.6)	57.4 (55.8–58.9)
Formerly Smoked	24.5 (23.4–25.7)	35.6 (30.0–41.2)	24.2 (23.1–25.3)
Current Smoker	18.6 (17.3–19.8)	23.8 (18.7–28.9)	18.4 (17.2–19.7)
Chronic Kidney Disease (CKD) **	14.1 (13.3–14.9)	39.6 (35.6–43.5)	13.3 (12.5–14.1)
**BMI**			
Normal Weight BMI < 25	26.2 (24.2–28.1)	19.5 (13.9–25.0)	26.4 (24.4–28.4)
Overweight BMI = 25–29.9	32.0 (30.6–33.4)	28.9 (22.1–35.7)	32.1 (30.7–33.5)
Obese BMI = 30–39.9	33.4 (31.5–35.3)	40.2 (33.5–47.0)	33.2 (31.3–35.1)
Morbidly obese BMI ≥ 40	8.5 (7.5–9.6)	11.4 (6.4–16.5)	8.4 (7.3–9.5)
Hypertension **	33.3 (31.7–34.8)	74.3 (70.1–78.6)	32.0 (350.5–33.5)
Age **	47.9 (0.29)	64.9 (0.71)	47.4 (0.29)
**Gender Females**	51.9 (51.1–52.7)	55.8 (50.6–61.0)	51.8 (50.9–52.7)
**Family Poverty–Income Ratio** (PIR < 1) **	14.3 (12.7–15.9)	20.5 (14.9–26.10)	14.1 (12.5–15.7)
Ethnicity **			
Non-Hispanic White	63.9 (60.1–67.8)	67.2 (61.0–73.4)	63.8 (60.0–67.7)
Non-Hispanic Black	11.4 (9.3–13.5)	15.2 (11.7–18.7)	11.3 (9.2–13.40
Hispanic	15.2 (12.5–18.0)	8.3 (6.4–10.6)	15.5 (12.7–18.2)
Other	9.4 (8.1–10.8)	9.3 (6.6–13.5)	9.4 (8.1–10.8)
Education Level **			
Some High School	15.1 (12.6–17.7)	22.2 (17.2–27.2)	14.9 (12.4–17.5)
High School Graduate	20.8 (18.8–22.8)	26.8 (20.3–33.4)	20.6 (18.5–22.8)
Some College or Above	64.1 (60.5–67.6)	51.0 (44.4–57.5)	64.4 (60.8–68.1)
Marital Status **			
Married	54.3 (52.3–56.3)	50.4 (42.2–56.6)	54.4 (52.4–56.5)
Widowed	5.9 (5.4–5.4)	19.0 (14.8–23.2)	5.5 (5.0–6.0)
Divorced	10.2 (9.5–10.9)	15.1 (11.0–19.2)	10.0 (9.3–10.8)
Separated	2.5 (2.2–2.9)	2.3 (1.3–3.9)	2.5 (2.2–2.9)
Never Married	18.5 (17.3–19.8)	8.7 (6.7–11.1)	18.8 (17.5–20.1)
Living With Partner	8.6 (7.8–9.5)	4.6 (3.0–7.0)	8.7 (7.9–9.7)
**All Deaths** (N, %) **	821 (3.8%)	118 (17.0%)	703 (3.4%)

Note: * *p* < 0.05 ** *p* < 0.01. Numbers with 95CI indicate 95% confidence intervals for proportions.

**Table 2:** All-cause mortality among adults aged 20 or older based on food insecurity and stroke.

	Total Population HR (95%CI)	Food Insecurity Only HR (95%CI)	Stroke Only HR (95%CI)	Stroke and Food Insecurity HR (95%CI)
**Stroke/Food Insecurity**	1.35 (0.79–2.32)	1.13 (0.63–2.03)	0.87 (0.39–1.94)	3.66 (1.64–8.14) **
**Smoking Status**				
Never Smoked (Ref.)	Ref	Ref	Ref	Ref
Formerly Smoked	1.62 (1.11–2.36) **	1.74 (1.12–2.70) *	1.47 (0.67–3.25)	0.95 (0.36–2.54)
Current Smoker	1.87 (1.17–3.00)	1.98 (1.12–3.50)	1.73 (0.94–3.17)	1.32 (0.39–4.46)
**Chronic Kidney Disease (CKD)**	2.39 (1.64–3.50) **	2.43 (1.68–3.52) **	2.47(1.62–3.78) **	2.77 (1.12–6.85) *
**Hypertension**	1.45 (1.01–2.07) *	1.52 (1.03–2.25) *	1.35 (0.82–2.22)	0.79 (0.40–1.57)
**Age**	1.07 (1.05–1.10) **	1.07 (1.05–1.10) **	1.09 (1.06–1.11) **	1.07 (1.03–1.12) *
**Gender (Ref. Female)**	1.70 (1.21–2.40) **	1.69 (1.17–2.44) **	2.20 (1.38–3.52) **	1.25 (0.37–4.21) *
**Family Poverty–Income Ratio** (Ref: PIR > 1)	2.54 (1.56–4.13) **	2.70 (1.43–5.08) **	2.72 (1.55–4.78) **	0.85 (0.25–2.90)
**Ethnicity**				
Non-Hispanic White	Ref	Ref	Ref	Ref
Non-Hispanic Black	0.66 (0.43–1.00)	0.69 (0.44–1.08)	0.64 (0.33–1.24)	0.31 (0.07–1.40)
Hispanic	0.71 (0.48–1.05)	0.71 (0.52–0.98)*	1.13 (0.68–1.90)	0.89 (0.20–4.05)
Other	1.28 (0.77–2.13)	1.36 (0.72–2.57)	1.35 (0.68–2.65)	0.49 (0.09–2.63)
**Education Level**				
Some College or Above	Ref	Ref	Ref	Ref
Some High School	0.91 (0.55–1.51)	0.98 (0.66–1.47)	0.74 (0.24–1.55)	0.46 (0.03–6.93)
High School Graduate	1.03 (0.67–1.60)	0.93 (0.61–1.43)	1.01 (0.56–1.84)	1.60 (0.53–4.85)
**Marital Status**	1.51 (0.99–2.31)	1.49 (0.97–2.28) *	1.69 (0.99–2.86)	2.67 (1.19–5.97) *

Note: * *p* < 0.05 ** *p* < 0.01. HR (95CI) indicates hazard ratios with 95% confidence intervals for the outcome (i.e., mortality). Ref indicates the reference group among each variable for comparison with other groups.

**Table 3:** All-cause mortality among male adults aged 20 or older based on food insecurity and stroke.

	Total Population HR (95%CI)	Food Insecurity Only HR (95%CI)	Stroke Only HR (95%CI)	Stroke and Food Insecurity HR (95%CI)
**Stroke/Food Insecurity**	0.97 (0.56–1.68)	0.74 (0.40–1.36)	0.74 (0.30–1.85)	7.20 (0.73–70.78)
**Smoking Status**				
Never Smoked (Ref.)	Ref	Ref	Ref	Ref
Formerly Smoked	1.48 (0.73–0.71)	1.56 (0.74–3.30)	1.30 (0.55–3.06)	0.34 (0.02–7.11)
Current Smoker	1.4 (0.53–4.19)	1.67 (0.50–5.61)	1.80 (0.69–4.71)	0.55 (0.01–33.01)
**Chronic Kidney Disease (CKD)**	2.36 (1.49–3.73) **	2.25 (1.51–3.35)	2.49 (1.52–4.26) **	2.92 (0.19–45.18)
**Hypertension**	1.25 (0.66–2.36)	1.31 (0.67–2.55)	1.29 (0.53–3.12)	0.73 (0.18 = 2.95)
**BMI**	1.00 (0.95–1.05)	1.00 (0.96–1.05)	1.02 (0.98–1.06)	0.85 (0.70–1.03)
**Age**	1.07 (1.04–1.10) **	1.07 (1.04–1.10) **	1.09 (1.06–1.11)	1.05 (1.00–1.11) *
**Family Poverty–Income Ratio** (Ref: PIR > 1)	2.94(1.82–5.35) **	3.33 (1.54–7.18) **	2.63 (115–6.05) *	0.58 (0.07–5.12)
**Ethnicity**				
Non-Hispanic White	Ref	Ref	Ref	Ref
Non-Hispanic Black	1.10 (0.55–2.22)	1.19 (0.60–2.39)	1.13 (0.57–2.23)	0.28 (0.05–1.59)
Hispanic	0.84 (0.47–1.53)	0.92 (0.52–1.61)	1.12 (0.56–2.25)	0.14 (0.01–3.11)
Other	0.95 (0.38–2.36)	0.92 (0.31–2.72)	1.07 (0.39–2.96)	0.41 (0.01–50.4)
**Education Level**				
Some College or Above	Ref	Ref	Ref	Ref
Some High School	0.72 (0.43–1.22)	0.66 (0.44–1.00)	0.89 (0.49–1.62)	1.21 (0.06–23.52)
High School Graduate	1.17 (0.46–2.96)	0.97 (0.33–2.89)	1.11 (0.31–4.00)	4.63 (0.60–35.91)
**Marital Status**	1.51 (0.77–2.95)	1.53 (0.70–3.36)	1.40 (0.65–3.02)	2.59 (0.23–28.94)

Note: * *p* < 0.05 ** *p* < 0.01. HR (95CI) indicates hazard ratios with 95% confidence intervals for the outcome (i.e., mortality). Ref indicates the reference group among each variable for comparison with other groups.

**Table 4:** All-cause mortality among female adults aged 20 or older based on food insecurity and stroke.

	Total Population HR (95%CI)	Food Insecurity Only HR (95%CI)	Stroke Only HR (95%CI)	Stroke and Food Insecurity HR (95%CI)
**Stroke/Food Insecurity**	2.33 (1.09–4.99)	2.03 (0.87–4.71)	1.36 (0.45–4.12)	8.76 (1.36–56.49) *
**Smoking Status**				
Never Smoked (Ref.)	Ref	Ref	Ref	Ref
Formerly Smoked	1.39 (0.61–3.16)	1.54 (0.57–4.11)	1.51 (0.45–5.05)	3.60 (0.09–145.76)
Current Smoker	2.35 (1.19–4.68) *	2.47 (1.13–5.41)	0.68 (0.12–3.78)	5.98 (0.02–1500.34)
**Chronic Kidney Disease (CKD)**	2.31 (1.12–4.75)	2.49 (1.28–4.84)	2.28 (1.18–4.42) *	7.04 (0.31–158.36)
**Hypertension**	1.29 (0.70–2.36)	1.18 (059–2.35)	1.03 (0.49–2.18)	7.61 (0.19–297.79)
**BMI**	1.03 (0.98–1.08)	1.03 (0.99–1.08)	1.00 (0.94–1.06)	0.91 (0.77–1.07)
**Age**	1.09 (1.06–1.11) **	1.09 (1.06–1.11)	1.09 (1.05–1.13)	1.07 (0.95–1.21)
**Family Poverty–Income Ratio** (Ref: PIR > 1)	1.61 (0.84–3.07)	1.53 (0.68–3.48)	1.94 (0.99–3.77)	0.14 (0.00–8.64)
**Ethnicity**				
Non-Hispanic White	Ref	Ref	Ref	Ref
Non-Hispanic Black	0.29 (0.18–0.47)	0.34 (020–0.56)	0.18 (0.03–1.21)	---
Hispanic	0.55 (0.26–1.18)	0.52 (0.23–1.21)	1.29 (0.45–3.68)	---
Other	1.59 (0.78–3/23)	1.85 (0.75–4.53)	1.70 (0.47–6.01)	---
**Education Level**				
Some College or Above	Ref	Ref	Ref	Ref
Some High School	1.40 (0.62–3.18)	1.87 (0.84–4.18)	0.79 (0.33–1.91)	0.04 (0.01–0.97) *
High School Graduate	1.14 (0.58–2.32)	1.13 (0.52–2.44)	1.19 (0.55–2.55)	5.72 (0.78–41.78)
**Marital Status**	1.57 (0.43–5.69)	1.45 (0.37–5.64)	2.56 (0.54–12.09)	21.62 (0.25–1864.94)

Note: * *p* < 0.05 ** *p* < 0.01. HR (95CI) indicates hazard ratios with 95% confidence intervals for the outcome (i.e., mortality). Ref indicates the reference group among each variable for comparison with other groups.

## Data Availability

The original contributions presented in this study are included in the article. Further inquiries can be directed to the corresponding author.
